# Exploring Self-Consciousness From Self- and Other-Image Recognition in the Mirror: Concepts and Evaluation

**DOI:** 10.3389/fpsyg.2019.00719

**Published:** 2019-05-07

**Authors:** Gaëlle Keromnes, Sylvie Chokron, Macarena-Paz Celume, Alain Berthoz, Michel Botbol, Roberto Canitano, Foucaud Du Boisgueheneuc, Nemat Jaafari, Nathalie Lavenne-Collot, Brice Martin, Tom Motillon, Bérangère Thirioux, Valeria Scandurra, Moritz Wehrmann, Ahmad Ghanizadeh, Sylvie Tordjman

**Affiliations:** ^1^Pôle Hospitalo-Universitaire de Psychiatrie de l’Enfant et de l’Adolescent (PHUPEA), Centre Hospitalier Guillaume Régnier, Université de Rennes 1, Rennes, France; ^2^Laboratoire de Psychologie de la Perception (LPP), Université Paris Descartes, CNRS UMR 8242, Paris, France; ^3^Laboratoire de Psychologie et d’Ergonomie Appliquées (LaPEA), Université Paris Descartes, UMR T7708, Boulogne Billancourt, France; ^4^Laboratoire de Physiologie de la Perception et de l’Action, Collège de France, CNRS UMR 7152, Paris, France; ^5^CHU de Brest – Service Hospitalo-Universitaire de Psychiatrie, CHU de Brest, Hôpital de Bohars, Bohars, France; ^6^Child and Adolescent Neuropsychiatry, University Hospital of Siena, Siena, Italy; ^7^Département de Neurologie, Centre de Mémoire de Ressource et de Recherche, CHU de Poitiers, Poitiers, France; ^8^Université de Poitiers, Unité de Recherche Clinique Intersectorielle en Psychiatrie à Vocation Régionale Pierre-Deniker du Centre Hospitalier Henri Laborit, Poitiers, France; ^9^INSERM U 1084, Experimental and Clinical Neurosciences Laboratory, Groupement de Recherche, CNRS 3557, Poitiers, France; ^10^Service Universitaire de Réhabilitation, Hôpital du Vinatier, Université Lyon 1, CNRS UMR 5229, Lyon, France; ^11^International Research Institute for Cultural Techniques and Media Philosophy, Bauhaus-Universität Weimar, Weimar, Germany; ^12^Department of Neuroscience, Research Center for Psychiatry and Behavioral Sciences, School of Advanced Medical Sciences and Technologies, Shiraz University of Medical Sciences, Shiraz, Iran

**Keywords:** self, self-consciousness, body-self, body image, body perception, intermodal sensory perception, body action, development

## Abstract

A historical review of the concepts of self-consciousness is presented, highlighting the important role of the *body* (particularly, body perception but also body action), and the *social other* in the construction of self-consciousness. More precisely, body perception, especially intermodal sensory perception including kinesthetic perception, is involved in the construction of a sense of self allowing self-other differentiation. Furthermore, the *social other*, through very early social and emotional interactions, provides meaning to the infant’s perception and contributes to the development of his/her symbolization capacities. This is a necessary condition for body image representation and awareness of a permanent self in a time-space continuum (invariant over time and space). Self-image recognition impairments in the mirror are also discussed regarding a comprehensive developmental theory of self-consciousness. Then, a neuropsychological and neurophysiological approach to self-consciousness reviews the role of complex brain activation/integration pathways and the mirror neuron system in self-consciousness. Finally, this article offers new perspectives on self-consciousness evaluation using a double mirror paradigm to study self- and other- image and body recognition.

## Introduction

Self-consciousness can be defined for an individual as the awareness of his/her own body in a time-space continuum and its interactions with the environment – including others. It also encompasses the awareness that the individual has of his/her own identity, built over time in interaction with others. It is at the root of higher level processes, such as the theory of mind or empathy, processes that allow us not only to be aware of others but also to differentiate ourselves from them, from their image and from their perceptive and emotional experiences (Decety and Sommerville, 2003; Rochat, 2003).

Self-consciousness is at the intersection of different disciplines, such as neurophysiology, psychiatry, psychology/neuropsychology, psychoanalysis and philosophy, which puts it at the center of many research topics. Many authors have highlighted the crucial role of the body in the development of self-consciousness, both as an interface with the environment and as an actual part of the self (body-self) (Damasio et al., 2000; Ionta et al., 2011). In addition, the importance of the mirror in psychoanalytic and psycho-developmental models of self-consciousness strengthens the place of body-self in the construction of self-image recognition that implies self-consciousness (Wallon, 1934).

We propose, in this article, first to conduct a historical review of research on the concepts of self-consciousness, including the development of self-consciousness and the role of the body (especially, body perception but also body action) and the *social other* in the construction of self-consciousness. In this perspective, neuropsychological and neurophysiological approaches to self-consciousness will be developed. Second, the importance of self-image and self-recognition in the mirror will be underlined, especially with regard to the interest of the mirror in the evaluation of self-consciousness.

## Concepts of Self-Consciousness and Self-Image

### Theoretical Bases: Conceptualizing the Self

For centuries, theorists have sought to understand and define self-consciousness (Maine de Biran, 1834; Piaget, 1936; Wallon, 1959b; Merleau-Ponty, 1964; Vygotsky, 1978; Neisser, 1991; Rochat, 2003). One of the theoretical models, developed especially by Piaget (1936) and Merleau-Ponty (1964), is that of an innate self-consciousness, at least in its bodily dimension. In this model, the subject is *born* subject and *knows himself* as a subject. His/her subsequent psychic development concerns how he/she will build and shape the world around him/her. According to Piaget (1936), the interactions between the child and the environment around him/her are governed by the rhythm at which the maturation of his/her central nervous system takes place. His fundamental work in developmental psychology has influenced many of his successors, including Merleau-Ponty (1964) whose philosophical work has partly focused on the phenomenology of perception. Some of the notions he developed joined Piaget’s ideas. Notably, he insisted on the importance of the other for the individual, while involving a certain degree of innate self-consciousness, especially with a body schema already present in the child at a very young age and thanks to which he/she will be able to interact with others.

At the beginning of the twentieth century different currents of thought appeared, principally developed by Vygotsky (1933). Vygotsky’s ideas were far from Piagetian theories, by questioning the Piagetian egocentric stage that places the child at the center of the development of the self and the surrounding world representations. For Vygotsky, the child is not the main worker of this construction; he/she is a learner and it is the presence of the other and of the external environment which will allow him/her to be able to build him/herself as an individual (Vygotsky, 1978).

These ideas were taken up by many contemporaries. In France, the influence of Wallon (1959b) was particularly important. Through his work, Wallon questions a possible co-construction of self-consciousness and consciousness of the other based on the individual’s interactions with the external environment. Wallon focuses on the importance of the presence of the other to be with the child in his/her self-construction. The child learns attitudes from others, first by simple mimicry and reciprocal emotional contagion. According to Wallon, this emotional reciprocity comes to sign the initial impossibility for the child to dissociate him/herself from others during the first months of life. He/she would not be aware of being a separate individual from his/her parents. It is through a game of reciprocal stimulation and alternation that the child finally would become aware of the boundary that separates him/her from the other, from his/her own *ego*. For Wallon, this development of self-consciousness ends at the age of 3, age of crisis in which the child can affirm him/herself and oppose to others his/her own desires and ideas. This approach dominated currents of thought during the following decades.

Neisser (1991) described two distinct ways of building the *self:* (a) Through body perceptions and interactions with the environment, and (b) Through the relationship with others. Furthermore, other authors such as Prinz (2013) discussed the notion of social mirroring and proposed that social mirroring is a prerequisite for the constitution of mental selves. Finally, Webb and Graziano (2015) highlighted the important role of attention processing in the development of self-consciousness with regard to external and internal environmental stimuli.

A summary of conceptualizations of the *self* is presented in Table 1.

**Table 1:** Conceptualizing the self.

Authors	Conceptualization
Piaget (1936)	The subject is *born* subject and *knows himself* as a subject. Psychological development concerns how children build and shape the world around, governed by the maturation of the central nervous system
Wallon (1959b)	The subject learns behaviors through imitation and emotional contagion and cannot be therefore dissociated from others during the first months of life. Through reciprocal stimulation and alternation, the child becomes aware of limits between him/herself and the other, and develops his/her own *ego*
Merleau-Ponty (1964)	Importance of the other. A certain degree of innate self-consciousness exists (body schema) allowing social interactions
Vygotsky (1978)	The subject learns from the others and the external environment how to build him/herself as an individual
Neisser (1991)	Two distinct ways of building the *self:* (a) through body perceptions and interactions with environmental objects, and (b) through relationships with others
Kiverstein (2007)	For each experience there is a neural representational system constituting the minimal supervenience basis for specific experiences


More global approaches to the concept of self-consciousness have been proposed more recently, notably by Damasio et al. (1999), whose work focuses on the study of the neural basis of cognition and behavior. He has been one of the most active researchers in the field of awareness/consciousness exploring the mechanisms that underlie it. In his work, he provides a summary of ideas developed over his various studies by offering three distinct levels of awareness/consciousness that ultimately lead to self-consciousness: (a) *Primary consciousness*, (b) *Reflexive consciousness*, and (c) *Self-consciousness.* Primary consciousness is a vigilant core consciousness which develops between the age of 6 months to 1 year old and allows the infant to evolve in his/her environment even when he/she is not able to differentiate between him/herself and the rest of the world. Primary consciousness is shared by other animal species with sensory organs and a complex brain (Jones and Mormede, 2002). Reflexive consciousness allows the individual to understand that he/she is the one who directs his/her own actions and thoughts, and that he/she controls his/her reasoning and behavior. It corresponds to the consciousness of not being the other and would be shared by humans and big primates. Self-consciousness, the higher level of consciousness, refers to the ability to appropriate one’s own history, to be aware of a unity of the self that persists despite the passage of time and the environmental changes. According to Damasio, this does not appear before the age of 2 in human beings. Interestingly, the age of 2 corresponds also to the child development of language.

The notion of primary consciousness developed by Damasio et al. (1999) can be compared to the concept of “minimal self.” The minimal self represents the most basic level of the self. It refers to self-consciousness as a subject of an immediate experience and pre-reflexive origin of action, experience and thought (Gallagher, 2000). The pre-reflexive “it is mine” (or feeling of belonging; of “mineness”) of a conscious experience, is a central characteristic of the minimal self. Therefore, the minimal self can be differentiated from more elaborate aspects of the self, such as the reflexive self (explicit consciousness of an “I”) and the narrative self (experience of a self with specific characteristics and one’s personal history).

More recently, Decety and Sommerville (2003) presented their conceptualization of *self* in a literature review. This conceptualization reflects in some ways the stratification model described by Damasio. Accordingly, the construction of the self is a multidimensional and evolving process that takes place from infancy and develops throughout the first years of life. This process involves physical, psychological and social factors and allows the development of different types of consciousness with different levels. Decety and Sommerville highlighted the cognitive dimension of self-development involving shared self-other representations, ultimately leading to self-other differentiation.

The different levels, types, contents and alterations of self- consciousness are summarized in Table 2.

**Table 2:** Levels, types, contents, and alterations of self-consciousness (based on Damasio et al., 1999; Decety and Sommerville, 2003; Rochat, 2003; Parnas and Henriksen, 2014; Keromnes et al., 2017).

	Consciousness	
Levels of consciousness^∗^	Pre-reflexive consciousness (implicit)	Early appearance, relies on bodily perception
	•*Level 1: Differentiation*	•*Relies on the experience of own bodily movements*
	•*Level 2: Situation*	•*Relies on intermodal sensory perception of the own body*
	Reflexive consciousness (explicit)	The self is expressed explicitly
	•*Level 3: Identification*	•*Identification of the self in the mirror*
	•*Level 4: Permanence*	•*Identification of a permanent self (invariant over time), in pictures and movies*
	Self-consciousness (explicit)	Later appearance, relies on mental representations
	•*Level 5: “Meta” self-awareness*	•*Notably, representations of how the child is perceived by others*
Types of consciousness	Agency	Consciousness of volition and ownership
	Distinctiveness	Consciousness of uniqueness
	Personal continuity	Consciousness of continuity through time
	Reflection	Consciousness of consciousness
Contents of consciousness	Physical	Physical features
	Active	Action skills
	Psychological	Traits and values
	Social/relational/collective	Social role and membership, reputation, relationship to others
Alterations of self-conscIousness	Presence	The sense of personal experience becomes affected
	Sense of Corporeality	Striking tendency to experience one’ s body predominantly as an object: an increasing experiential distance between subjectivity and corporeality (“disembodiment”)
	Stream of Consciouness	Mental contents become quasi-autonomous (“automatic” thoughts), without ipseity and with a rupture of the stream of thoughts (thoughts may appear as if from nowhere)
	Self-demarcation	Inferential reflection arises as a consequence of a deficient sense of myness
	Solipsism and existential reorientation	To be excessively preoccupied with philosophical, supernatural, or metaphysical themes


*^∗^Five levels (Rochat, 2003) in contrast to a level zero corresponding to a level of confusion with absence of self-consciousness.*

In light of these different approaches, two primordial concepts can be highlighted. The first is that the acquisition of self-consciousness comes mainly by the differentiation between oneself and the other, with the recognition of each other’s identities. The second concept is that the body, an interface between oneself and the other, is one of the essential keys in the course of this process. This concept is developed in the next section.

Concerning the first concept, self-consciousness would be built in relation to the other (relational dyad), through relational and emotional synchronization, and through the other’s eyes (Wallon, 1984; Feldman, 2007; Haag et al., 2005, 2010). The mother looks at the baby and the baby looks at the mother, but he/she sees also his/her reflection in the eyes of the mother. Self-consciousness, with the integration of body image, also passes by the imitation of the other, going for example from simple imitation in the new born of sticking out the tongue to more complex development including the child’s verbal language (Nadel-Brulfert and Baudonnière, 1982; Nadel et al., 1983; Nadel, 2011).

Here we could hypothesize that self-consciousness is built up through the imitation of the other, with the representation of what is identical through synchronization, but also with the representation of what is different. Later, the appearance of a gendered body refers to sexual differentiation and is probably in adolescence a new mobilizing lever of this process.

### The Role of the Body in Self-Consciousness

#### Body Perception and Self-Consciousness

Today, links between body and mind seem to be well established. Clinical practice recalls it every day with regard to the frequency of the psychosomatic manifestations observed in patients with psychiatric disorders (Testa et al., 2013). The body appears here as reflecting psychological problems and at the center of the psychological process of self-consciousness (Gernet, 2007).

For Wallon (1959a), when the child is born, he/she sees him/herself as dislocated, with distinct parts and members, and gradually, he/she sees them unified in a coherent body. The consciousness of a body self would be an indispensable prerequisite for the construction of the child’s personality. This concept was first introduced in 1794 under the name of *cenesthesia*. Hübner defined *cenesthesia* as a general sensibility that represents to the soul the state of its body whereas the sensibility informs the soul on the external world and the internal sense gives representations, judgments, ideas and concepts (Starobinski, 1977). Wallon will later describe it in a simpler way by designating under this term two types of sensibilities: an internal and visceral sensibility, and a proprioceptive and postural sensibility whose joint action will be responsible for kinesthetic sensations.

This concept of *cenesthesia* evolved toward the concept of *body schema* at the end of the nineteenth century, following Bonnier’s (1904) clinical observations on impairments in the perception of the own body resulting from certain neurological lesions or benign disorders such as vertigo. The existence of a normal perceptive system to which these anomalies would be related is then hypothesized. This perceptual system would correspond to what Head and Holmes et al. (1911–1912) conceptualized shortly thereafter under the term of *body schema*. This corporeal pattern would gradually form in the first months of life, when the child seems to have a keen interest in exploring his/her own body through touch, but also to explore his/her immediate environment. Through this exploration, the child gradually learns the boundaries between his/her body and his/her surroundings. Although several studies have underlined the risk of body schema deficits in children with severe visual deficits (for a review, see Lueck and Dutton, 2015), Head and Holmes rejected any participation of the optical pathways in the acquisition of this body schema which reflects an overall intuition concerning the present situation of the body in space.

This last remark emphasizes the fundamental difference between this body schema concept and the self-image concept introduced by Schilder (1968). The first one is based on postural elements whereas the other refers to the symbolic and affective experience based above all on a visual perception of oneself. The two, however, are not dissociated given that they contribute together to the constitution of *the body-self*.

As body schema, self-image is not innate but is acquired gradually. The concept of self-image is affectively and symbolically charged. Self-image is not just an observed image. It includes also how individuals represent their own body in their mind and the others’ representations on their own body. This representation is initially performed as part of the interactions between the child and the other, as if the child could see him/herself first through the eyes of the other before being able to imagine his/her own body. Various authors have described the important role of the mirror in the construction of self-image and, more broadly, the self. This aspect will be detailed later.

This overview of ideas which were developed for over a hundred years shows the importance of the body in major theories of developmental psychology. Indeed, more recent authors, such as Damasio et al. (1999), have highlighted the central place occupied by the body in the phenomenon of consciousness. They support the idea that conscious thinking is primarily based on our visceral perceptions. In their model, they developed different possible levels of self-consciousness, placing bodily perception before any level of consciousness. The perception of the external world described in primary consciousness becomes possible only if this fundamental bodily perception is operational.

In recent years, many authors have also participated in improving our understanding of the pathways involved in self-consciousness, with a very specific focus on “body self-consciousness” (Aspell et al., 2009; Pasqualini et al., 2013). This concept is divided into three dimensions: self-localization, first-person perspective, and self-identification.

Different experimental studies have shown that bodily self-consciousness is malleable. Sforza et al. (2010), for example, studied facial recognition in healthy individuals, through a simple experimental paradigm. An examiner touched the subject’s face while the subject observed the same action being applied simultaneously to another person’s face. The results of this study showed frequent errors in identifying the image of the other as his own. Similarly, by manipulating visual-tactile inputs, an illusory feeling of ownership can be induced by an artificial hand (rubber hand illusion; Botvinick and Cohen, 1998). As a matter of fact, viewing another person’s hand or face being stroked in synchrony with strokes applied to our own corresponding non-visible hand or face can induce illusory self-attribution of the visible hand or face. Moreover, participants perceive their hand to be at a position that is displaced toward the fake hand’s position (proprioceptive drift) or judge another person’s face as similar to their own face. Many experimental paradigms have shown similar results, suggesting that bodily self-consciousness may waiver with contradictory sensory stimulation. In addition, as discussed below, our motor actions may also contribute to self-consciousness.

#### Body Action and Self-Consciousness

As recently underlined, the motor control system in the brain not only controls complex actions but is also concerned by body representation (Murata et al., 2016). More precisely, Murata et al. (2016) showed that the motor control system contributes to perception of the hands as part of one’s own body. According to Gallagher (2005), the perception of one’s own body is the fundamental process of self-recognition. In this way, the hands are not only effectors in movement, but could be considered as a link between the mind and motor control. Along those lines, what is called now the sense of agency can be viewed as a subjective awareness that one’s generated action is attributed to one’s self. The sense of agency occurs when an executed action is recognized as being generated by one’s own body. The sense of agency is thus expected to occur exclusively during voluntary movement. According to Blakemore et al. (1999), a copy of the motor command, that is the efference copy, can pass into the forward model to predict feedback in response to a given motor command. In this way, the comparison between sensory feedback and the corollary discharge contributes both to the precision of the movement and to recognition of who generated the observed action. In turn, the sense of agency participates to the construction of self-consciousness through the production and the control of motor actions.

Several neuropsychological and neuroimaging experiments have revealed that the inferior parietal cortex is involved in the sense of agency (for a review, see Murata et al., 2016). Indeed, as demonstrated by Sirigu et al. (1999), patients with lesions of the inferior parietal cortex present deficits in agency recognition. Moreover, in the same way, a few human brain imaging studies have reported that the inferior parietal cortex is involved in the detection of agency of action in healthy participants (Farrer et al., 2003, 2008; Decety and Grèzes, 2006; Chambon et al., 2013). Interestingly as we will discuss below, parietal lesions are also responsible for spatial neglect as well as somatoparaphrenia, two neuropsychological deficits that can also affect self-consciousness.

### Neuropsychological Approach to Self-Consciousness

#### Disturbed Sense of Agency and Disturbed Body Ownership

As discussed above, the sense of body ownership, as well as the awareness of being causally involved in an action, the sense of agency, have been mostly investigated in healthy participants by using experimental as well as functional neuroimaging methods (Farrer et al., 2003; Tsakiris et al., 2007). A complementary approach is the study of neurological patients showing specific neuropsychological disturbances of these senses after brain damage.

As discussed below, brain-damaged patients, especially after a stroke, may present anosognosia for hemiplegia (AHP) affecting the sense of ownership and therefore self-consciousness. Brain-damaged patients with AHP deny typically the weakness of their paretic or plegic contralesional limb and are convinced that they move properly. These patients may also show a disturbed sense of ownership, with respect to paretic/plegic limb. They experience their contralesional limb as not belonging to them and may even attribute them to other people. This deficit is often called ‘somatoparaphrenia’. AHP is thus characterized by their false belief that they are not paralyzed. Their feeling of being or not being causally involved in an action – their sense of agency – is thus dramatically disturbed. As incredible as it may appear, despite the obvious fact that the contralesional limb is severely paralyzed, these patients behave as if the disorder did not exist. When they are asked to move the paretic/plegic arm or leg, they may do nothing or may move the limb of the opposite side. However, in both situations, they are either convinced that they have successfully executed the task or may argue that they can move in a generic manner. Interestingly, although they are unable to move their contralesional limb when asked to do so, they may explain their impossibility either by confabulations (I could move it yesterday, but my arm is now tired) or by external causes (the ground is slippery, and I cannot walk on it) (Nathanson et al., 1952). Regarding the neuro-anatomical correlates of the AHP, several studies have suggested that the right insular cortex might be a crucial anatomical region in integrating input signals related to self-awareness about the functioning of body parts (for a review, see Karnath and Baier, 2010). In addition, confirming this hypothesis, converging evidence has been reported that the anterior insular cortex is also a central structure for pain mechanisms and temperature regulation (Craig et al., 1996; Kong et al., 2006). In this way, the anterior insular cortex could well represent an important correlate of human “interoception” as well as a crucial cortical area for body ownership, for the sense of agency and more generally for self-consciousness (Craig, 2002, 2009). Moreover, the anterior insular cortex was suggested to be involved in other cognitive and emotional processes that could well contribute to self-consciousness such as the feelings of anger or anxiety (Phillips et al., 1997; Damasio et al., 2000), craving (Contreras et al., 2007; Naqvi et al., 2007), and visual self-recognition (Devue et al., 2007).

Finally, anosognosia and disturbed sense of body ownership are often associated with another neuropsychological deficit consecutive to a right parietal lesion and affecting spatial representation: unilateral spatial neglect (USN).

#### Personal Neglect

Unilateral spatial neglect is a disorder in which patients are unaware of the hemispace contralateral to the lesion. Usually a left USN is observed after a right parietal lesion. As Schilder (1968) has proposed, space can be divided in extrapersonal space, peripersonal space, and personal space. Along those lines, patients suffering from USN may ignore either the extrapersonal space (either near or far) or the personal space contralateral to the lesion. In this latter case, when suffering from personal neglect, patients ignore their own contralesional body parts. Indeed, patients may not use their contralesional hemibody although not being paralyzed. In some cases, patients may exhibit somatophrenia and can explain that their contralesional arm or leg is behind the closet, or that their husband or wife took it with them. Importantly, many patients are unaware they have these problems (anosognosia).

A neuropsychological approach can indeed be of interest to assess the role of the different cortical and subcortical structures involved in self-consciousness to decipher the neurophysiological basis of self-consciousness as presented in the next section.

### Neurophysiological Approach to Self-Consciousness

Evolutionary psychology postulates that self-consciousness, as well as other higher cognitive faculties, would be unique to the human being and thus, would distinguish us from animal species, even from the most evolved ones (Rochat, 2018). These theories are now challenged by neurobiological advances, highlighting the involvement of certain brain structures in the process of self-consciousness reported in some primates.

#### Complex Activation and Integration Pathways

Schilder, psychiatrist and psychoanalyst, departed from psycho-developmental theories of self-consciousness in 1935 to question the neurophysiological mechanisms allowing an individual to be situated in a given space-time (Schilder, 1968). His ideas opened new perspectives on various research projects. Since that time and still today, many experimental paradigms have been designed and developed to better understand the neurological pathways of self-consciousness.

Lhermitte (1939) was one of the first to publish his research on the neurophysiological mechanisms of self-consciousness. He described a probable activation of right parietal cerebral structures related to the process of acquisition of the self-image. Numerous studies supported later this hypothesis and it appears nowadays well established that right brain structures, particularly parietal ones, are involved in global self-consciousness (Taylor, 2001).

More specifically, besides the involvement of the inferior parietal cortex in the sense of agency described above in the “*Body action and self-consciousness*” section, the primordial role of the temporoparietal junction has been also reported (Ionta et al., 2011; Graziano, 2018). It corresponds to a zone of integration of multimodal sensory information that may play a key role in the first-person perspective, and the distinction between oneself and the other, as well as in some more complex mechanisms of the theory of mind, which includes the ability to understand the intentions, desires, and beliefs of the other. Aspell et al. (2012) studied more particularly its activation during the phenomenon of out-of-body experiences both by continuous electroencephalographic monitoring during such phenomena, but also by observing that such experiments could be triggered in healthy individuals by transcranial magnetic stimulation (TMS) of the temporoparietal junction (Blanke et al., 2005).

The role of frontal cortical structures was also discussed concerning more specifically an activation of the pre-frontal cortex which would intervene in the process of differentiation between self and others (Van Veluw and Chance, 2014). It is noteworthy that several fRMI studies on the theory of mind have highlighted the key role of the median prefrontal cortex (Van Veluw and Chance, 2014).

Finally, the role of the vestibular system was also described in the development of spatial bodily self-consciousness (Pfeiffer et al., 2014). One of its functions is to provide information on the position of the body taking into account the variations of the Earth’s gravitational system, essential for the brain’s encoding of the body’s spatial orientation in the environment. Some studies have hypothesized that the vestibular system could be part of a larger network involved in spatial exploration including the parietal lobes and the anterior insular cortex already mentioned in the section on “*Disturbed sense of agency and disturbed body ownership*” (Brandt et al., 1994; Karnath et al., 2004). This could well explain how caloric vestibular stimulation may reduce somatophrenia. Indeed, it was demonstrated that such stimulation applied in right brain-damaged patients can induce transitory remission of anosognosia for hemiparesis as well as permanent disappearance of somatophrenia (Cappa et al., 1987; Bisiach et al., 1991; Rode et al., 1992; Vallar et al., 2003). Also, caloric vestibular stimulation can reduce AHP and USN (Cappa et al., 1987; Bisiach et al., 1991; Rode et al., 1992; Vallar et al., 2003), confirming the role of the vestibular-parietal network in body awareness and self-consciousness.

It is noteworthy that integrated models of self-consciousness, involving sensory and motor multimodal integration, are related to ideas already developed by Sherrington almost one century ago. Sir C.S. Sherrington (1906) was an English neurologist who received the Nobel prize in medicine with Adrian (1932) for their work on the neural system. According to their work, the self-consciousness in the here and now is based on visuo-musculo-labyrinthic or tactile-muscular-labyrinthic perceptions (Wallon, 1959a). This model echoes also some of Piaget’s ideas, as he described within his developmental stages a first sensorimotor stage during which the child exists only through movement and sensation (Piaget, 1956). This description shows even today the importance of sensory stimulation and how it can be integrated in the brain toward the construction of self-consciousness, especially in very young children.

Within this process of integration and complex activation, the role of a particular neuron system – the mirror neurons – is subject of much debate.

#### The Mirror Neuron System

Mirror neurons were first described by Rizzolatti and Sinigaglia (2008). They are a system of motor neurons whose particularity is to activate themselves both when we perform a given action but also when we see someone else performing the same action, or even when we think of or speak about its realization without however, initiating it. They were first detected in monkeys after it was observed that they could frequently perform an action immediately after seeing it in one of their congeners, as if mirroring the other.

These descriptions might suggest that the neurons involved in such reactions are at the level of the optical pathways and are activated by visual stimulation. However, Rizzolatti’s studies in functional brain imaging suggest that this “mirrored” reaction would correspond to a brain activation at the level of the premotor frontal cortex, the superior temporal sulcus, and certain parietal areas (Rizzolatti and Sinigaglia, 2008). Other recent functional MRI studies confirm these data, without showing any activation in the occipital visual areas (Calvo-Merino et al., 2005). Studies of mirror neurons system in primates showed an activation of the F5 brain area that corresponds in humans to the Broca area (i.e., the inferior part of F3 corresponding to Brodmann’s areas 44 and 45). It could be hypothesized that these mirror neurons have also a role in the production of verbal language and in the ability to communicate with others.

It is recognized that these neurons underlie partly our ability to connect with each other. This finding placed them at the center of social cognition. Their role was particularly discussed in the ability to differentiate oneself from the other, but also in the interactions with others, at bodily, affective, and cognitive levels (as in the phenomenon of empathy, for example).

A parallel can be made here between the mirror neurons functioning and the gestual and emotional reciprocity described by Wallon. In the Wallon theory (1959b), the infant reproduces many actions that he/she sees in adults (a smile, for example) as if he/she was facing a mirror. It is through these experiences, more precisely through identification and differentiation processes, that self-consciousness can develop.

At present, the mirror neuron system suffers from some criticism for its lack of specificity and more recent research suggests that it has been granted quickly with too much credit (Hickok, 2014). Their discovery nevertheless created an emulation that has brought a lot to scientific research.

Despite constant efforts to better understand the specific paths involved in self-consciousness, these are not yet well established. Limits appear given the complexity of the information to be processed. For some authors such as Damasio et al. (1999), Tononi and Edelman (2000), or Seth et al. (2006), there is no fixed structure responsible for the existence of self-consciousness at its different levels. For these authors, self-consciousness responds in fact to a much more comprehensive brain activation that allows an individual to locate him/herself in the here and now, and to take into consideration his/her personal history with associated affects.

## Self-Image and the Mirror

### A Brief History of the Mirror

Today, omnipresent in our homes, the mirror is an ancient object whose form or use has changed over the centuries. The appearance of first mirrors is difficult to date. Descriptions can be found in ancient times when they were already known for their reflective capacity. The first mirrors were made of glass and lead. Their preparation from these materials involve some work and so made the object scarce and expensive. It remained the richest privilege for centuries. In the middle ages, for example, some most precious materials such as gold or silver, could be used to manufacture mirrors (Melchior-Bonnet, 2011).

It was not until the end of the seventeenth century that mirrors were more affordable, spread in homes, and became a need for everyone. The introduction of mirrors in our lives has probably much changed the way people perceive their own image (Melchior-Bonnet, 2011).

Alongside these historical and societal considerations, it is interesting to note that the mirror has long been seen as a fascinating object. In the study of the Inca civilization, we find mirror descriptions used as “fire lighter” (Nordenskiôld, 1926). This very special ability, yet based on simple optical properties, made it an almost magical object and, in fact, a luxury item. In the Middle Ages, the most beautiful and expensive mirrors were installed in castles of noble or royal people, and poets or storytellers of the time were celebrating their magical power of reflection. It is precisely this power of fascinating reflection that is found in many ancient myths. Since ancient times, self-reflection is the subject of many productions. The most common is the myth of Narcissus or Perseus. In both cases, the mirror or the reflect leads to a tragic death. Narcissus pays the price for his vanity and love for his reflection he sees in the water. Perseus is using the reflectivity of a mirror to defeat and kill his enemy Medusa. It might be seen here as the beginning of a reflection on self-consciousness.

### The Importance of the Other in the Construction of Self-Consciousness: The Theories of Self-Recognition in the Mirror

In his work on building self-consciousness, Wallon (1934) explains how young children, interacting with their environment, gradually become aware of their *own body*. One of the major parts of his work is the description of the reaction of the child in the mirror. Wallon observed that between 6 months and 2 years of age, the child develops a fascination with his/her reflection in the mirror, even after having understood that is fictional. The child can contemplate this reflection a long time, enjoying looking at this “other” who is not “a real self.” Starting with the first reflections, looking at the mirror with his/her parents, he/she turns to them in search of reassurance, as a validation that the image he/she is seeing is actually him/herself.

As we mentioned earlier, it is through this game of alternation and sharing between him/herself and the other that the child becomes aware of his/her individuality. The mirror is, for Wallon, one of the major mediators in this process, because it is through it that the child can interact with others.

Zazzo (1948) followed some of Wallon’s ideas. He published his observations from the reactions of an infant confronted with his/her images via his/her reflection in a mirror, but also photographs and films. He states that when the child is facing the mirror as well as other visual aids, the recognition of the other is far ahead of self-recognition. He establishes also that for the three types of images (mirror, photo, and film) there is a first period during which we observe that the subject appears not to recognize or even watch his/her own image. Besides, he explains that the recognition of the other through the mirror begins well before in a picture or movie. However, if the self-image in the mirror is recognized much earlier, it remains a long time affected by uncertainty and anxiety unlike still images such as photographs.

The finding showed that between 2 and 3 years of age, the child becomes increasingly aware of his/her body image and would be able to understand that he/she is alone facing his/her own image and that his/her reflection is not somebody else or a double. According to Zazzo, this development occurs in parallel with the explosion of language, an additional tool that helps the child to understand the distinction between him/herself and the other and between him/her and his/her reflection.

Yet the essential work of Wallon and Zazzo appear neglected when, some years later, Lacan grabbed the topic and brought it to psychoanalysis by publishing “*The mirror stage as formative of the function of the I as it is revealed in psychoanalytic experience*.” In a replay of the theories developed by Wallon, Lacan describes how the mirror stage helps the child to give up his/her fragmented body to become aware of its identity through the mirror image (Lacan, 1966), the description of the “*Stage of the mirror*” by Lacan is now a cornerstone of the psychoanalytic approach to the construction of the self. Lacan himself referred the origin of his theory to the work of the American psychologist James Mark Baldwin who influenced also Piaget and Vygotsky. Baldwin’s theories focused on the progressive distinction between self and other through social interactions. He was one of the first to see the way the child behaves in front his/her reflection as an indicator of the construction of the self (Müller and Runions, 2003).

Many other authors were concerned about this topic in the aftermath of Lacan, notably Françoise Dolto, who published in 1987 with Nasio “*The child of the mirror*.” If she adhered partially to Lacan’s theories, her approach differs on an essential point. Unlike Lacan (and Wallon before him) who described the infant as a fragmented being, with no containment in the months preceding the identification with reflection, Dolto highlights a primary narcissism that makes the child a cohesive whole, maintained through basic external, and visceral sensations. Therefore, the infant cannot be fully satisfied with this image as it is incomplete, reflecting only one side of his/her body, whereas he/she even “feels as a whole in his/her being” (Dolto and Nasio, 2002).

The weight given to the mirror in psychoanalytic and psycho-developmental theories stresses the importance of body-self and self-image, but also the importance of the other in the construction of self-consciousness.

### Impairments in Self-Image Recognition in the Mirror

Disorders of self-consciousness are related to various disturbances of the pathways involved in self-consciousness. Different components of self-consciousness can be impaired and produce various clinical syndromes. Impairments in bodily self-consciousness lead to somatognosic disorders among which are found the out-of-body experiences, the heautoscopy, and the autoscopic hallucinations. These phenomena have been widely studied in the case of disorders of neurological origin, particularly in certain dementia syndromes (Blanke, 2007). Their mechanisms are still poorly understood, although Blanke has been able to demonstrate a dysfunction of the temporoparietal areas previously described, with the additional involvement of certain occipital areas in the case of autoscopic hallucinations (Blanke and Mohr, 2005).

Heautoscopy was described by Lhermitte (1939) in his work «*The image of our body*» like an almost hallucinatory experience during which the patient suddenly sees his/her image appearing in front of him/her. He explains it schematically by two essential components: a visual hallucination and a disturbance of bodily self-consciousness. The latter causes the individual a feeling of partial depersonalization that makes it difficult for him/her to locate him/herself – either in his/her own body or projected in the autoscopic image. One of the variations of this heautoscopy (or illusion of the double) is the phenomenon of negative heautoscopy that Maupassant has described so well, when sinking into madness: Indeed, he described this phenomenon in which the individual no longer sees his/her image in a reflective surface. This illustration highlights an example of a situation in which the relationship to the mirror is disturbed. It shows also that such disruptions in the recognition of self-image are not confined to neurological disorders. They are also common in psychiatric disorders and especially psychotic disorders.

It is currently accepted that early disturbances of the child’s psychological development can be associated with various psychiatric disorders which may eventually become fixed in adulthood (Jones, 1997; Tackett et al., 2009). Given the important role of the mirror during psychological development, we can suppose that the relation to the mirror, and therefore to the reflection, is disturbed in children with atypical psychological development. Salem Shentoub was one of the first to study self-recognition in children with developmental disabilities, including intellectual disability. He described the reaction of “mentally retarded” children in front of the mirror and observed differences in their behaviors compared to typically developing children (Rustin et al., 1954). The reported reactions are varied, ranging from an apparent absence of self-image recognition to complex affective manifestations, including preliminary interactions with the reflection or various stereotyped behaviors. However, the children’s reactions in front of the mirror appear to be an extension of their usual behavior and the confrontation with their image does not trigger in these children more specific reactions, even in the most severe cases. The most alarming observation was the apparent absence of self-recognition, which questions a possible absence of recognition of the other as well as self-consciousness impairments. Nevertheless, Shentoub observed that the repetition of mirror experiences in the same child allowed him/her to become acquainted with the other and with his/her image, and this was associated with an overall positive behavioral evolution. He was already raising here a possible remediation that could be accomplished through the mirror image.

These intellectually disabled children observed by Shentoub did not show a psychotic disorder marked by an internal disorganization or a rupture with reality. However, it cannot be ruled out that young patients with schizophrenia might also exhibit some atypical behaviors when facing mirrors due to their own perceptual and/or cognitive developmental disturbances.

The psychoanalytic theories presented briefly in this article allow us to consider these disturbances of self-image recognition as an indicator or even a possible marker of a disturbance of psychological development occurring upstream of the mirror stage. Lacan, himself, mentions in his work the absence of reaching the mirror stage in psychotic patients, thus preventing their symbolic identification with their own image.

On this topic, Abely (1930) described in the early 1930s “a need for certain individuals to examine themselves at length and frequently in front of a reflective surface” (quoted in Meaulle, 2007). He reported here a phenomenon he observed at the dawn of the appearance of early dementia in some of his patients. In his descriptions, this fixation on the mirror can be accompanied by a search for dialogue with the reflection which is considered as a distinct other. Shortly after him, Delmas (1929) published similar observations.

The observations Abely and Delmas developed, almost simultaneously but separately, now refer to the same concept, the “mirror sign.” This sign would be for them a clinical marker of psychotic disorganization. The mirror sign, for these authors, more than a fascination for the patient’s reflection, corresponds in fact to a search for the patient’s own image in the mirror – an image that disintegrates more and more permanently with the onset of psychotic disorders. It is noteworthy that in the interpretation they make of this mirror sign, this sign would be part of the prodromal phase, and would disappear once the disorder developed.

It is noteworthy that the mirror can be used to study impairments in self-image recognition. A new double mirror paradigm for the study of self-other differentiation, self-identity and self-image recognition, and manipulation of spatial reference frames in social interactions, was proposed by Alain Berthoz (Collège de France, Paris), using the “Double Mirror” designed by Moritz Werhmann (Alter Ego System that includes a set of white computer-controlled light emitting diodes/LEDs fixed on the frame of the mirror on both sides), and studied in healthy participants by Thirioux et al. (2016). Previous studies (Harrington and Spitzer, 1989; Caputo et al., 2012; Bortolon et al., 2017) have used the mirror to explore self-image recognition in schizophrenia but the Alter Ego System (c), which combines the facial images of two individuals sitting on each side of the mirror, offers a new double mirror paradigm to examine self-other recognition impairments in individuals with schizophrenia. Self-other recognition impairments have been also examined in schizophrenia by Slowinski et al. (2017) but they used a “mirror game” (without a real mirror) based on interactions between the patient and an artificial agent, a computer avatar or a humanoid robot, which cannot be compared to self-other recognition involving only human individuals. The paradigm of the double mirror was used for the first time to study self-other differentiation in individuals with schizophrenia compared to typically developing controls (TDC) (Keromnes et al., 2018). The visual recognition task consisted in recognizing more the other’s face through the mirror (as through a transparent window) or his/her own face reflected in the mirror according to the light intensity of the LEDs set (the higher the light intensity is, the more visible is the image). The results showed that individuals with schizophrenia, independently of age and schizophrenia severity, were centered on their own image, with both significant earlier self-recognition, and delayed other-recognition compared to TDC during the visual recognition task. In addition, there was no significant effect of intermodal sensory stimulation (visual-tactile or visual-kinesthetic stimulation) on self–other recognition in individuals with schizophrenia, whereas self-centered functioning was significantly increased by visual–tactile stimulation and decreased by visual–kinesthetic stimulation in TDC. The findings suggest that self–other recognition impairments might be a possible endophenotypic trait of schizophrenia. It would be of interest to conduct the same experimental study using the double mirror on individuals with childhood onset schizophrenia and catatonia, characterized by a very early onset of schizophrenia but also severe clinical impairments and longer episodes of schizophrenia (Bonnot et al., 2008), to verify if similar results are observed in this population.

## Conclusion

The literature review presented in this article emphasizes the role of body perception, body actions and of the self-image in the construction of self-consciousness. Of importance, we demonstrated here that a multidisciplinary approach is mandatory to address such a complex concept. We aimed also to highlight the interest of self-image recognition in the mirror to assess self-consciousness but also the role of the other in self-image recognition. Self-image development might be a good indicator of the evolution of the self-consciousness process, especially through self-and other-image recognition in the mirror (Tordjman and Maillhes, 2009). Self-consciousness can be impaired in one or several of its components (identity, body, etc.). Self-recognition, and notably self-image recognition, can be disturbed in various disorders, especially neurodevelopmental disorders (dementia, psychiatric disorders, etc.) (Blanke, 2007). Considering impairments in self-consciousness and self-image recognition may open important perspectives, especially for early diagnosis and therapeutic strategies in neurodevelopmental disorders. However, a limit of such phenomenological inquiry remains the detection of these disturbances that relies on patients’ verbal reports (Martin et al., 2014). These patients’ reports should indeed be interpreted with caution, especially because body-self is related to non-verbal aspects of consciousness. Thus, a challenge consists in finding a way to objectify such self-disturbances in individuals with a non-verbal approach (Mishara et al., 2014). The double mirror, mentioned previously in this article, might be a useful instrument to investigate further self–other recognition impairments in self-consciousness disorders in general and neurodevelopmental disorders such as schizophrenia or Autism Spectrum Disorder. Self–other face identification in the mirror may improve bodily self-consciousness and sustain self–other differentiation in these disorders. Future studies are required to explore this perspective. In particular, the double mirror system could be useful for early diagnosis, follow-up, and therapeutic perspectives based on cognitive remediation helping individuals with self-consciousness disorders to improve self–other differentiation.

## Author Contributions

GK and ST wrote the first draft of the article. SC contributed in a significant way to this article by adding notably the part on the neuropsychological approach to self-consciousness. M-PC revised the first draft of the article. AB, MB, RC, FDB, NJ, NL-C, BM, TM, BT, VS, MW, and AG reviewed and approved the final version of the article.

## Conflict of Interest Statement

The authors declare that the research was conducted in the absence of any commercial or financial relationships that could be construed as a potential conflict of interest.
